# Supplementary material for: Comparison of Transcriptomic Analysis of the Conjunctiva in Glaucoma-Treated Eyes with Dry Eyes and Healthy Controls
Source: Biomolecules. 2023 Dec 25;14(1):30. doi: 10.3390/biom14010030 (PMC10813521; doi:10.3390/biom14010030)
Supplement: Supplementary file 1 [file biomolecules-14-00030-s001.zip › biomolecules-2675775-supplementary.pdf]

biomolecules

MDPI

Supplementary Materials:

Table S1 Frequency and combination of medications used in eyes included in RNA sequencing. PGA: prostaglandin analogues. BB: beta-blockers. CAIs: carbonic anhydrase inhibitors. AA: alpha agonists.

PGA-BB-CAI-AA	Frequency	Percentage
1-1-0-0	9	27.3
0-1-1-0	5	15.2
1-1-1-0	5	15.2
0-0-0-1	3	9.1
0-1-0-1	3	9.1
0-1-0-0	2	6.1
0-1-1-1	2	6.1
1-1-1-1	2	6.1
1-0-0-0	1	3.0
1-1-0-1	1	3.0
**Total**	**33**	**100.0**

Table S2. List of 60 genes altered in the conjunctiva of treated patients compared to healthy conjunctiva by RNA sequencing.

ID gene	Gene Name	log Fold Change	p Value	GO Molecular Function	GO Biological Process
**ENSG00000106278.11**	*PTPRZ1*	1.893	0.002	Transmembrane receptor protein tyrosine phosphatase activity	Regulation of oligodendrocyte progenitor proliferation
**ENSG00000114013.15**	*CD86*	1.770	0.001	Signaling receptor binding and coreceptor activity	Immune system
**ENSG00000112799.8**	*LY86*	1.548	0.001	Sensor component	Immune system
**ENSG00000095970.16**	*TREM2*	1.523	0.003	Scaffold protein binding	Amyloid-beta clearance and immune system
**ENSG00000175857.8**	*GAPT*	1.484	0.005	Scaffold protein binding	Immune system
**ENSG00000140678.16**	*ITGAX*	1.471	0.009	Signaling receptor activity	Cell adhesion and immune system
**ENSG00000043591.5**	*ADRB1*	1.446	0.004	Nucleotide binding regulatory protein-coupled receptor	Adrenergic receptor signaling pathway and response to cold, fear.
**ENSG00000010671.15**	*BTK*	1.415	0.002	Non-membrane spanning protein tyrosine kinase activity	Immune system
**ENSG00000136869.14**	*TLR4*	1.409	0.000	Amyloid-beta binding and LPS sensor activity	Immune system

**ENSG00000186818.12**	*LILRB4*	1.336	0.001	Antigen binding and signaling receptor activity	Immune system
**ENSG00000169896.17**	*ITGAM*	1.326	0.005	Cargo receptor activity	Cell adhesion and immune system
**ENSG00000011600.11**	*TYROBP*	1.311	0.000	Signaling receptor binding	Actin cytoskeleton organization and immune system
**ENSG00000158869.10**	*FCER1G*	1.196	0.010	IgE receptor activity	Immune system and mast cell activation
**ENSG00000000938.12**	*FGR*	1.195	0.006	Fc-γ receptor I complex binding	Immune system
**ENSG00000101336.13**	*HCK*	1.187	0.004	Tyrosine kinase activity and signaling receptor binding	Cell adhesion and immune system
**ENSG00000104903.4**	*LYL1*	1.175	0.000	Transcription factor activity	B cell differentiation and blood vessel maturation
**ENSG00000157017.15**	*GHRL*	1.170	0.001	Hormone activity	Adult feeding behavior
**ENSG00000153071.14**	*DAB2*	1.167	0.008	GTPase activator activity	Cellular response to LPS, TNF and cellular response to unfolded protein
**ENSG00000175899.14**	*A2M*	1.166	0.007	Endopeptidase inhibitor activity	Extracellular matrix disassembly
**ENSG00000213203.2**	*GIMAP1*	1.163	0.000	GTP binding activity	UK
**ENSG00000038945.14**	*MSR1*	1.159	0.008	Cargo receptor activity	Phagocytosis, engulfment
**ENSG00000134516.16**	*DOCK2*	1.139	0.002	T-cell receptor binding	Actin remodelling and phagocytosis
**ENSG00000137491.14**	*SLCO2B1*	1.134	0.006	Organic anion transmembrane transporter activity	Solute transport membranes
**ENSG00000203747.10**	*FCGR3A*	1.092	0.007	IgG binding	Immune system
**ENSG00000100097.11**	*LGALS1*	1.091	0.000	Carbohydrate binding	Apoptotic processand cellular response to glucose stimulus
**ENSG00000204482.10**	*LST1*	1.086	0.002	Leukocyte specific transcript 1	Cell morphogenesis and immune system
**ENSG00000155926.13**	*SLA*	0.968	0.003	Signaling receptor binding	Cell differentiation and regulation of cell population proliferation
**ENSG00000079215.13**	*SLC1A3*	0.955	0.003	Glutamate binding	Response to light stimulus and response to wounding
**ENSG00000136286.15**	*MYO1G*	0.954	0.004	Actin and calmodulin binding	Cell gliding and cell-substrate adhesion
**ENSG00000250312.7**	*ZNF718*	0.896	0.009	DNA binding and metal ion binding	Regulation of transcription and DNA-templated
**ENSG00000264230.8**	***ANXA8L1***	**-1.153**	**0.009**	Calcium-dependent phospholipid binding	Endosome organization
**ENSG00000156414.18**	*TDRD9*	-1.159	0.003	RNA helicase activity	Cell differentiation and gene silencing
**ENSG00000135318.11**	*NT5E*	-1.162	0.008	Nucleotide binding	Leukocyte cell-cell adhesion and negative regulation of inflammatory response
**ENSG00000181449.3**	*SOX2*	-1.226	0.004	Transcription activator activity	Cell differentiation and cell fate commitment
**ENSG00000164627.17**	*KIF6*	-1.266	0.002	Microtubule binding	Microtubule-based movement
**ENSG00000132429.9**	*POPDC3*	-1.272	0.004	cAMP binding	Tissue development and differentiation
**ENSG00000128578.9**	*STRIP2*	-1.289	0.000	Striatin interacting protein	Cell migration and cytoskeleton organization
**ENSG00000118971.7**	*CCND2*	-1.302	0.010	Cyclin-dependent protein serine/threonine kinase	Cell division

**ENSG00000141314.12**	*RHBDL3*	-1.344	0.001	Calcium ion binding and endopeptidase activity	UK
**ENSG00000110975.8**	*SYT10*	-1.373	0.009	Clathrin binding	Exocytosis
**ENSG00000164949.7**	*GEM*	-1.446	0.006	GTPase activity and calcium channel regulator activity	Immune system and mitotic cell cycle
**ENSG00000203710.11**	*CR1*	-1.452	0.001	Complement component	Complement activation
**ENSG00000119508.17**	*NR4A3*	-1.467	0.004	Steroid hormone receptor activity	Cellular response to catecholamine and corticotropin-releasing hormone stimulus
**ENSG00000135046.13**	*ANXA1*	-1.469	0.004	Cadherin binding and single-stranded RNA binding	Immune system and inflammatory response
**ENSG00000142149.8**	*HUNK*	-1.469	0.002	Protein serine/threonine kinase activity	Signal transduction
**ENSG00000128422.16**	*KRT17*	-1.469	0.006	Structural constituent of cytoskeleton and MHC class II protein binding	Cornification and epidermis development
**ENSG00000137440.4**	*FGFBP1*	-1.480	0.004	Fibroblast growth factor binding	Regulation of fibroblast growth factor receptor signaling pathway
**ENSG00000164687.10**	*FABP5*	-1.494	0.001	Lipid binding and retinoic acid binding	Epidermis development
**ENSG00000135919.12**	*SERPINE2*	-1.504	0.006	Signaling receptor binding	Secretion by cell and sensory perception
**ENSG00000182950.2**	*ODF3L1*	-1.547	0.001	UK	UK
**ENSG00000153234.13**	*NR4A2*	-1.570	0.000	Steroid hormone receptor activity	Cellular response to catecholamine and corticotropin-releasing hormone stimulus
**ENSG00000160223.17**	*ICOSLG*	-1.604	0.002	Signaling receptor binding	Immune system
**ENSG00000112276.13**	*BVES*	-1.711	0.001	Structural molecule activity	Epithelial cell-cell adhesion
**ENSG00000163735.6**	*CXCL5*	-1.733	0.004	Chemokine activity	Immune system
**ENSG00000100234.11**	*TIMP3*	-1.871	0.001	Metalloendopeptidase inhibitor activity	Cellular response to organic substance and negative regulation of metalloendopeptidase activity
**ENSG00000123243.14**	*ITIH5*	-1.903	0.001	Endopeptidase inhibitor activity	Collagen-containing extracellular matrix
**ENSG00000183833.16**	*MAATS1*	-2.061	0.001	Cilium movement	UK
**Ensg00000275993.2**	*Sik1b*	-2.070	0.000	Serine/threonine kinase activity	Intracellular signal transduction and protein phosphorylation
**Ensg00000136244.11**	*Il6*	-2.103	0.003	Cytokine binding	Immune system
**Ensg00000183092.16**	***Begain***	**-2.204**	**0.000**	**Kinase activity**	**Neuropathic pain and interaction at synapses**

Table S3. List of 60 genes most significantly altered in the conjunctiva of dry eye patients compared to healthy conjunctiva by RNA sequencing.

ID gene	Gene Name	log Fold Change	p Value	GO Molecular Function	GO Biological Process
**ENSG00000090104.11**	*RGS1*	2.779	0.001	GTPase activator activity and calmodulin binding	G protein-coupled receptor signaling pathway and inmune response
**ENSG00000147604.13**	*RPL7*	2.355	0.001	Nucleic acid binding and protein homodimerization activity	RNA catabolic process, nonsense-mediated decay
**ENSG00000144199.11**	*FAHD2B*	2.259	0.000	Hydrolase activity mitochondria	ROS formation in respiratory chains
**ENSG00000186847.5**	*KRT14*	2.174	0.007	Structural constituent of cytoskeleton	Aging and cornification
**ENSG00000169413.2**	*RNASE6*	2.155	0.002	Nucleic acid binding and ribonuclease activity	Innate immune response and RNA catabolic process
**ENSG00000123560.13**	*PLP1*	2.125	0.005	Major myelin protein	Astrocyte development and inflammatory response
**ENSG00000197410.13**	*DCHS2*	2.052	0.002	Calcium ion binding	Homophilic cell adhesion via plasma membrane adhesion molecules
**ENSG00000198712.1**	*MT-CO2*	1.993	0.006	Copper ion binding and cytochrome-c oxidase activity	Mitochondrial electron transport
**ENSG00000198189.10**	*HSD17B11*	1.962	0.001	Steroid dehydrogenase activity	Androgen catabolic process and estrogen biosynthetic process
**ENSG00000198727.2**	*MT-CYB*	1.848	0.007	Metal ion binding and cytochrome-c oxidase activity	Mitochondrial electron transport and response to stress and ion
**ENSG00000087076.8**	*HSD17B14*	1.831	0.009	Steroid dehydrogenase activity	Androgen catabolic process and estrogen biosynthetic process
**ENSG00000136160.15**	*EDNRB*	1.786	0.009	Endothelin receptor activity	Aging, proliferation and pain reception
**ENSG00000150594.6**	*ADRA2A*	1.753	0.005	Adrenergic receptor activity	Proliferation and wound healing
**ENSG00000158869.10**	*FCER1G*	1.698	0.001	IgE receptor activity	Immune system and mast cell activation
**ENSG00000269028.3**	*MTRNR2L12*	1.667	0.002	Receptor antagonist activity	Negative regulation of execution phase of apoptosis
**ENSG00000113140.10**	*SPARC*	1.551	0.009	Extracellular matrix binding	Proliferation, migration, and cell differentiation
**ENSG00000131153.8**	*GINS2*	1.529	0.000	DNA duplex unwinding	DNA replication
**ENSG00000091136.13**	*LAMB1*	1.527	0.004	Extracellular matrix structural constituent and integrin binding	Cell adhesion and proliferation
**ENSG00000165568.17**	*AKR1E2*	1.505	0.006	Oxidoreductase activity	Oxidation-reduction process
**ENSG00000089685.14**	*BIRC5*	1.499	0.005	Chaperone binding	Cell proliferation and preventing apoptosis
**ENSG00000276600.4**	*RAB7B*	1.496	0.004	GTPase activity	inflammation and vesicle trafficking
**ENSG00000115041.12**	*KCNIP3*	1.491	0.003	Calcium ion binding	Intracellular protein transport and apoptosis
**ENSG00000124664.10**	*SPDEF*	1.464	0.008	DNA-binding transcription factor activity	Goblet cells differentiation
**ENSG00000011028.13**	*MRC2*	1.436	0.004	Collagen binding and transmembrane signaling receptor activity	Collagen catabolic process and endocytosis
**ENSG00000176153.11**	*GPX2*	1.430	0.003	Electron transfer activity	Cellular response to oxidative stress
**ENSG00000136522.13**	*MRPL47*	1.428	0.001	Structural constituent of ribosome	Mitochondrial translation
**ENSG00000150201.14**	*FXYD4*	1.395	0.004	ATPase binding	Ion channel regulator activity
**ENSG00000163453.11**	*IGFBP7*	1.360	0.005	Insulin-like growth factor binding	Cell adhesion and response to retinoic acid

**ENSG00000198677.11**	*TTC37*	1.320	0.006	Catabolism of deadenylated mRNA	RNA catabolic process
**ENSG00000135547.8**	*HEY2*	1.305	0.003	DNA-binding transcription factor activity	Embryonic development
**ENSG00000107201.9**	*DDX58*	-0.748	0.008	Double-stranded RNA binding and RNA helicase	Innate immune response
**ENSG00000137492.7**	*THAP12*	-0.758	0.000	Transcription factor activity	Negative regulation of cell population proliferation
**ENSG00000155090.14**	*KLF10*	-0.760	0.004	Transcription factor activity	Negative regulation of cell population proliferation
**ENSG00000135049.15**	*AGTPBP1*	-0.775	0.006	Metallocarboxypeptidase activity and tulin binding	Eye photoreceptor cell differentiation and mitochondrion organization
**ENSG00000135317.12**	*SNX14*	-0.781	0.006	Phosphatidylinositol-3.5-bisphosphate binding	Autophagosome maturation
**ENSG00000152601.17**	*MBNL1*	-0.786	0.001	Double-stranded RNA binding	Myoblast differentiation and nervous system development
**ENSG00000154114.12**	*TBCEL*	-0.788	0.008	Alpha-tubulin binding	Microtubule cytoskeleton organization
**ENSG00000114480.12**	*GBE1*	-0.803	0.003	Glucan branching enzyme activity	Carbohydrate metabolic process
**ENSG00000165322.17**	*ARHGAP12*	-0.807	0.001	GTPase activator activity	Actin filament organization and morphogenesis of an epithelial sheet
**ENSG00000196263.7**	*ZNF471*	-0.826	0.010	DNA binding and metal ion binding	Regulation of transcription
**ENSG00000115339.13**	*GALNT3*	-0.827	0.007	Calcium ion binding and carbohydrate binding	Carbohydrate metabolic process and fibroblast growth factor receptor signaling pathway
**ENSG00000140450.8**	*ARRDC4*	-0.831	0.005	Protein binding. bridging involved in substrate recognition for ubiquitination	Extracellular vesicle biogenesis and positive regulation of ubiquitin-protein transferase activity
**ENSG00000119938.8**	*PPP1R3C*	-0.845	0.008	Glycogen binding	Glycogen biosynthetic process
**ENSG00000180530.10**	*NRIP1*	-0.853	0.006	Hormone receptor binding	Regulation of transcription
**ENSG00000150457.8**	*LATS2*	-0.853	0.004	ATP binding Source and metal ion binding	Cell division and hippo pathway
**ENSG00000179941.7**	*BBS10*	-0.885	0.006	ATP binding and RNA polymerase II repressing transcription factor binding	Photoreceptor cell maintenance and visual perception
**ENSG00000239305.6**	*RNF103*	-0.898	0.004	E3 ubiquitin-protein ligase	Central nervous system development
**ENSG00000096717.11**	*SIRT1*	-0.901	0.009	NAD-dependent histone deacetylase activity	Angiogenesis and response to damage
**ENSG00000261210.7**	*CLEC19A*	-0.910	0.004	Carbohydrate binding	UK
**ENSG00000196542.8**	*SPTSSB*	-0.949	0.000	Serine palmitoyltransferase	Lipid metabolic process
**ENSG00000185947.14**	*ZNF267*	-0.956	0.006	DNA-binding transcription factor activity	UK
**ENSG00000105855.9**	*ITGB8*	-0.965	0.010	Extracellular matrix protein binding	Cell adhesion and migration
**ENSG00000176208.8**	*ATAD5*	-0.976	0.000	ATP binding and DNA binding	Regulation of mitotic cell cycle phase transition
**ENSG00000273604.1**	*EPOP*	-1.177	0.001	Chromatin binding	Regulation of transcription
**ENSG00000148516.21**	*ZEB1*	-1.273	0.007	DNA-binding transcription factor activity	Embryonic camera-type eye morphogenesis
**ENSG00000169282.17**	*KCNAB1*	-1.342	0.005	Voltage-gated ion channels subunit	Oxidation-reduction process
**ENSG00000050344.8**	*NFE2L3*	-1.409	0.005	DNA-binding transcription factor activity	Positive regulation of transcription
**ENSG00000107249.22**	*GLIS3*	-1.413	0.005	DNA binding and metal ion binding	Transcription factor involved in eye development
**ENSG00000183092.16**	*BEGAIN*	-1.720	0.004	Kinase activity	Neuropathic pain and interaction at synapses
**ENSG00000154548.8**	*SRSF12*	-1.872	0.000	Nucleic acid binding	Regulation of mRNA splicing

Table S4. List of 60 genes most significantly altered in the conjunctiva of dry eye patients compared to healthy conjunctiva by RNA sequencing.

ID gene	Gene Name	Log FC	*p* Value	GO Molecular Function	GO Biological Process
ENSG00000187242.5	*KRT12*	2.505	0.0078	Structural molecule activity	Cornification and keratinization
ENSG00000154548.8	*SRSF12*	1.866	0.0000	Nucleic acid binding	Regulation of mRNA splicing
ENSG00000179636.14	*TPPP2*	1.599	0.0050	Tubulin binding	Cell differentiation
ENSG00000106278.11	*PTPRZ1*	1.527	0.0072	Transmembrane receptor protein tyrosine phosphatase activity	Regulation of oligodendrocyte progenitor proliferation
ENSG00000043591.5	*ADRB1*	1.435	0.0066	Nucleotide binding regulatory protein-coupled receptor	Adrenergic receptor signaling pathway and response to cold, fear.
ENSG00000050344.8	*NFE2L3*	1.400	0.0020	DNA-binding transcription factor activity	Positive regulation of transcription
ENSG00000238083.7	*LRRC37A2*	1.378	0.0059	Leucine-rich repeat-containing protein	UK
ENSG00000136867.10	*SLC31A2*	1.371	0.0034	Copper ion transmembrane transporter activity	Cellular copper ion homeostasis
ENSG00000114346.13	*ECT2*	1.312	0.0045	GTPase activator activity	Bicellular tight junction assembly and cell morphogenesis
ENSG00000105327.17	*BBC3*	1.224	0.0002	Cysteine-type endopeptidase activity<br>involved in apoptotic process	Cellular response to damage
ENSG00000058866.14	*DGKG*	1.185	0.0069	Diacylglycerol kinase<br>activity Source: BHF-UCL	Glycerolipid metabolic process
ENSG00000188051.6	*TMEM221*	1.158	0.0024	Non-membrane spanning protein tyrosine kinase activity	Immune system
ENSG00000010671.15	*BTK*	1.150	0.0093	Transcription factor activity	Cell differentiation
ENSG00000123095.5	*BHLHE41*	1.145	0.0002	Transcription factor activity	Animal organ morphogenesis and animal organ morphogenesis
ENSG00000104154.6	*SLC30A4*	1.136	0.0020	Zinc ion transmembrane transporter activity	Zinc ion homeostasis and response to toxic substance
ENSG00000196542.8	*SPTSSB*	1.132	0.0000	Ceramide biosynthetic process serine C-palmitoyltransferase activity	Sphingolipid metabolism and in Lipid metabolism
ENSG00000164440.14	*TXLNB*	1.104	0.0083	Syntaxin binding	UK
ENSG00000114805.17	*PLCH1*	1.098	0.0009	Calcium-dependent phospholipase C activity	Lipid catabolic process and release of calcium ion into cytosol
ENSG00000241106.7	*HLA-DOB*	1.091	0.0084	MHC class II protein complex binding	Immune system
ENSG00000183018.8	*SPNS2*	1.034	0.0007	Sphingolipid transporter activity	Lipid transport, immune cells homeostasis and regulation of eye pigmentation
ENSG00000105855.9	*ITGB8*	1.028	0.0018	Extracellular matrix protein binding	Cell adhesion and migration
ENSG00000074527.11	*NTN4*	1.027	0.0060	Laminin-1 binding	Substrate adhesion-dependent cell spreading and tissue development
ENSG00000113328.18	*CCNG1*	1.021	0.0089	Cyclin-dependent protein serine/threonine kinase	Cell division

ENSG00000104043.14	*ATP8B4*	0.999	0.0046	ATPase-coupled intramembrane lipid transporter activity	Golgi organization and neutrophil degranulation
ENSG00000239305.6	*RNF103*	0.997	0.0004	Ubiquitin protein ligase activity	Endoplasmic reticulum mannose trimming and protein ubiquitination
ENSG00000273604.1	*EPOP*	0.965	0.0030	Chromatin binding	Regulation of transcription
ENSG00000137878.17	*GCOM1*	0.950	0.0006	Intracellular signal transduction	UK
ENSG00000115339.13	*GALNT3*	0.869	0.0011	Carbohydrate binding	Protein O-linked glycosylation via threonine
ENSG00000145375.7	*SPATA5*	0.867	0.0016	ATPase activity	Cell differentiation
ENSG00000198157.10	*HMGN5*	-1.629	0.0005	Chromatin and RNA binding	Chromatin organization
ENSG00000112769.18	*LAMA4*	-1.645	0.0024	Extracellular matrix structural constituent	Cell adhesion and extracellular matrix organization
ENSG00000107165.12	*TYRP1*	-1.732	0.0040	Oxidoreductase activity	Regulation of melanin biosynthetic process
ENSG00000135919.12	*SERPINE2*	-1.746	0.0015	Serine-type endopeptidase inhibitor activity	Innervation and secretion by cell
ENSG00000150201.14	*FXYD4*	-1.756	0.0000	ATPase binding and ion channel regulator activity	Ion transmembrane transport
ENSG00000206072.12	*SERPINB11*	-1.773	0.0038	Serine-type endopeptidase inhibitor activity	Negative regulation of endopeptidase activity
ENSG00000228253.1	*MT-ATP8*	-1.818	0.0001	Proton transmembrane transporter activity	Mitochondrial ATP synthesis coupled proton transport
ENSG00000197641.11	*SERPINB13*	-1.819	0.0000	Cysteine-type endopeptidase inhibitor activity	Negative regulation of keratinocyte apoptotic process
ENSG00000173114.12	*LRRN3*	-1.841	0.0009	UK	UK
ENSG00000198739.10	*LRRTM3*	-1.844	0.0072	Protein-protein interactions	UK
ENSG00000118402.5	*ELOVL4*	-1.862	0.0023	Fatty acid elongase activity	Fatty acid biosynthetic process
ENSG00000128422.16	*KRT17*	-1.874	0.0007	Structural constituent of cytoskeleton and MHC class II protein binding	Cornification and epidermis development
ENSG00000136522.13	*MRPL47*	-1.893	0.0000	Structural constituent of ribosome	Mitochondrial translation
ENSG00000206075.13	*SERPINB5*	-1.901	0.0000	Serine-type endopeptidase inhibitor activity	Extracellular matrix organization and regulation of epithelial cell proliferation
ENSG00000210082.2	*MT-RNR2*	-1.945	0.0000	Receptor antagonist activity and signaling receptor binding	Apoptotic process and cellular iron ion homeostasis
ENSG00000145423.4	*SFRP2*	-1.941	0.0038	Endopeptidase activator and fibronectin binding activity	Apoptotic process and negative regulation of epithelial cell proliferation
ENSG00000119508.17	*NR4A3*	-1.948	0.0001	Transcription activator activity	Cellular respiration and mast cell degranulation
ENSG00000198189.10	*HSD17B11*	-2.038	0.0000	Steroid dehydrogenase activity	Steroid metabolic process
ENSG00000142149.8	*HUNK*	-2.080	0.0000	Protein serine/threonine kinase activity	Signal transduction
ENSG00000138653.9	*NDST4*	-2.095	0.0012	Heparan sulfate N-acetylglucosaminyltransferase activity	Heparin biosynthetic process

ENSG00000198712.1	*MT-CO2*	-2.116	0.0006	Copper ion binding and cytochrome-C oxidase activity	Mitochondrial electron transport
ENSG00000136160.15	*EDNRB*	-2.117	0.0001	Endothelin receptor activity	Aging, proliferation, and pain reception
ENSG00000198677.11	*TTC37*	-2.118	0.0000	Catabolism of deadenylated mRNA	RNA catabolic process
ENSG00000169429.10	*CXCL8 (IL8)*	-2.257	0.0001	Chemokine activity	Immune response
ENSG00000137440.4	*FGFBP1*	-2.379	0.0000	Fibroblast growth factor binding	Cell-cell signaling
ENSG00000164687.10	*FABP5*	-2.563	0.0000	Lipid binding and retinoic acid binding	Epidermis development
ENSG00000135046.13	*ANXA1*	-2.799	0.0000	Cadherin binding and single-stranded RNA binding	Immune system and inflammatory response
ENSG00000198727.2	*MT-CYB*	-2.836	0.0000	Metal ion binding and cytochrome-c oxidase activity	Mitochondrial electron transporter and response to stress and ion
ENSG00000169413.2	*RNASE6*	-2.941	0.0000	Nucleic acid binding and ribonuclease activity	Innate immune response and RNA catabolic process
ENSG00000090104.11	*RGS1*	-2.996	0.0000	GTPase activator activity and calmodulin binding	G protein-coupled receptor signaling pathway and immune response
ENSG00000147604.13	*RPL7*	-3.125	0.0000	Nucleic acid binding and protein homodimerization activity	RNA catabolic process, nonsense-mediated decay

Fig. S1A

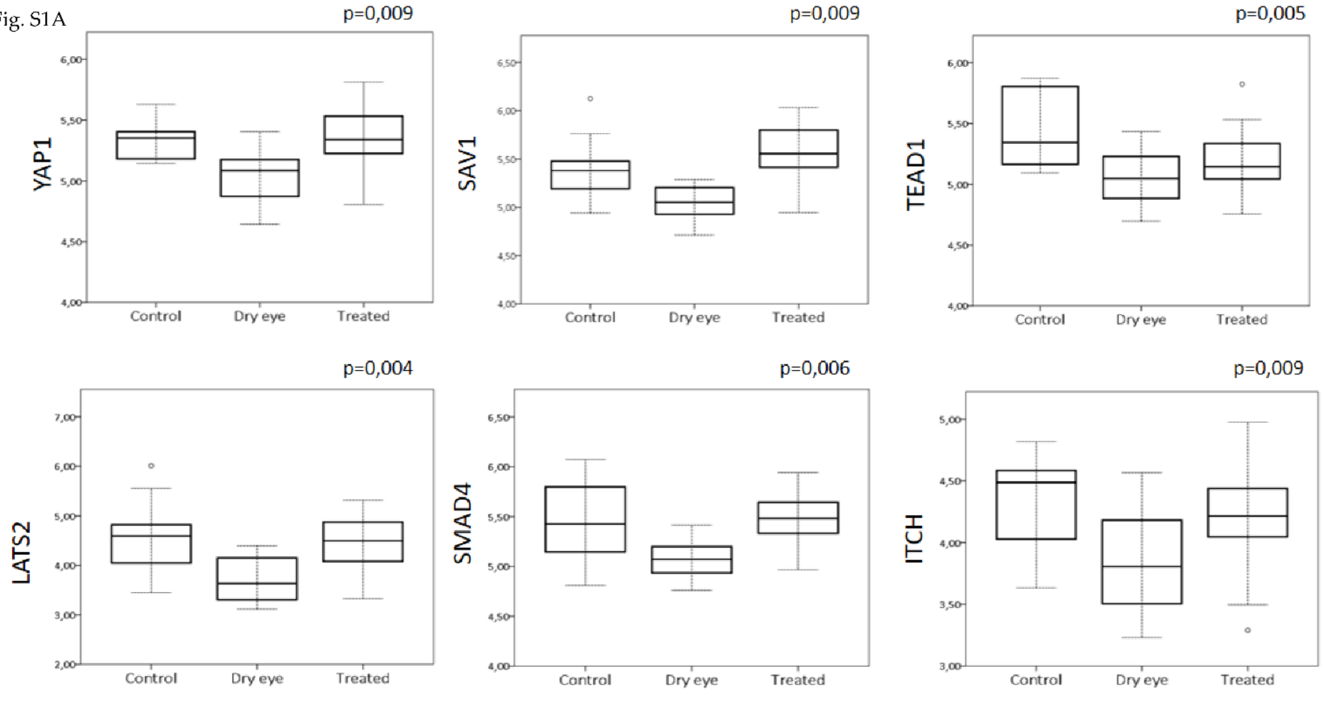

Fig. S1B

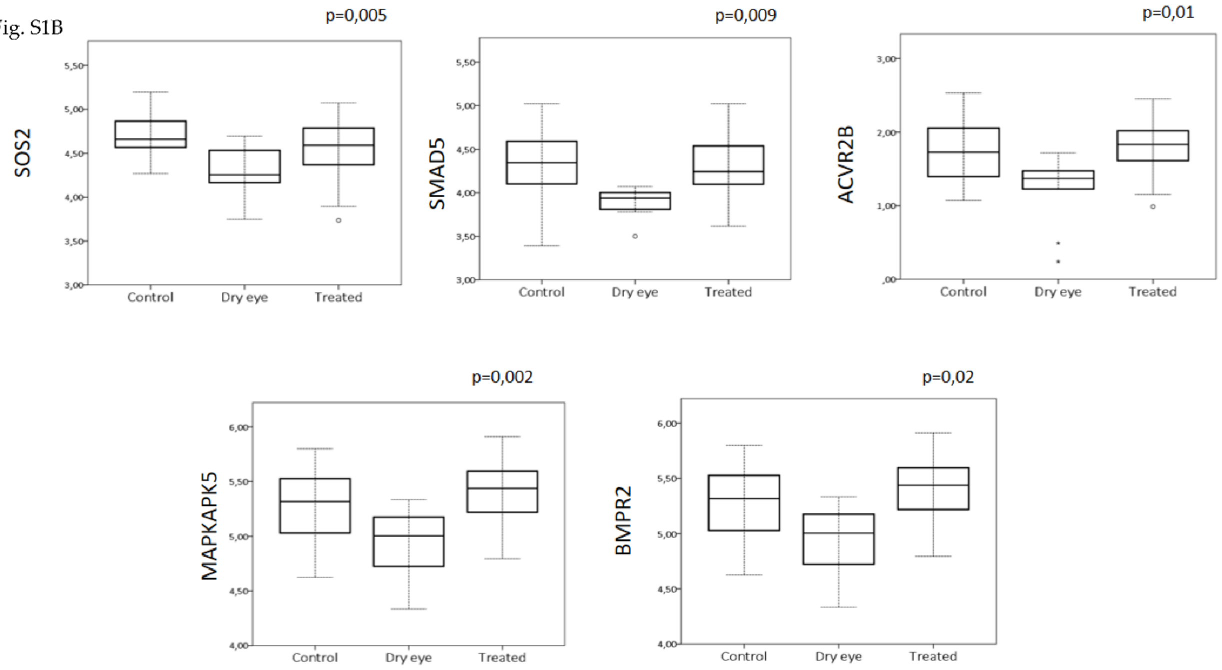

Fig. S1C

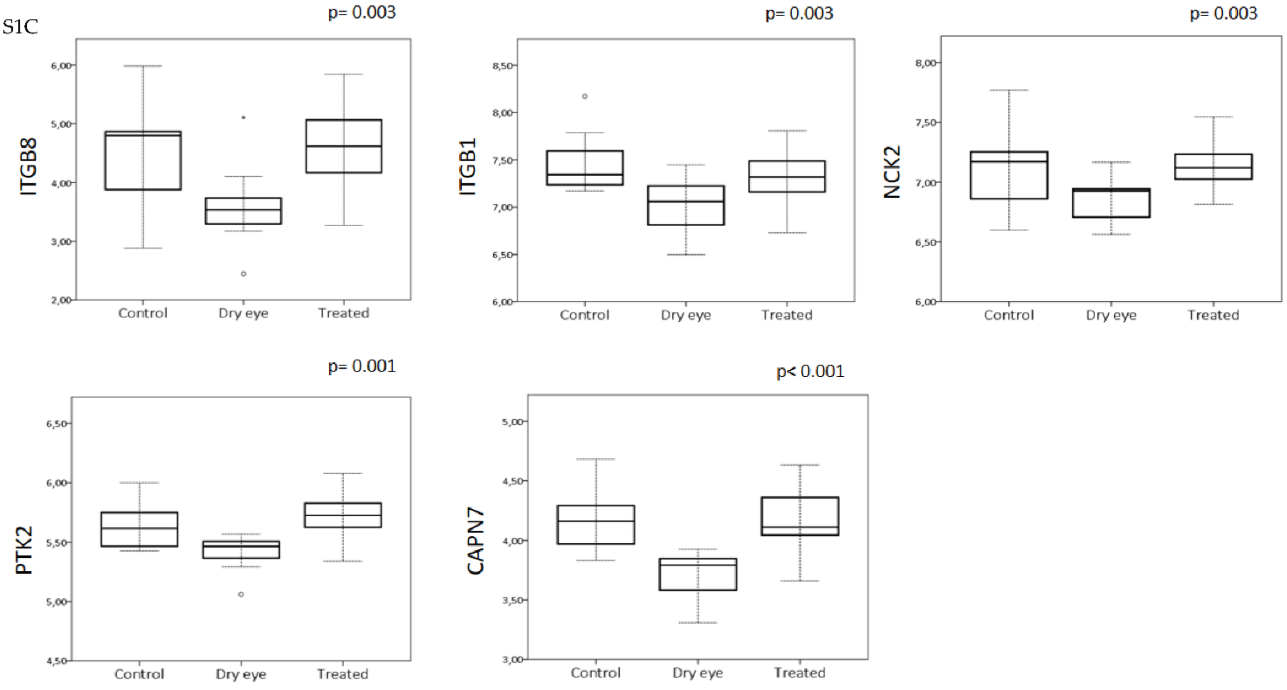

Fig. S1D

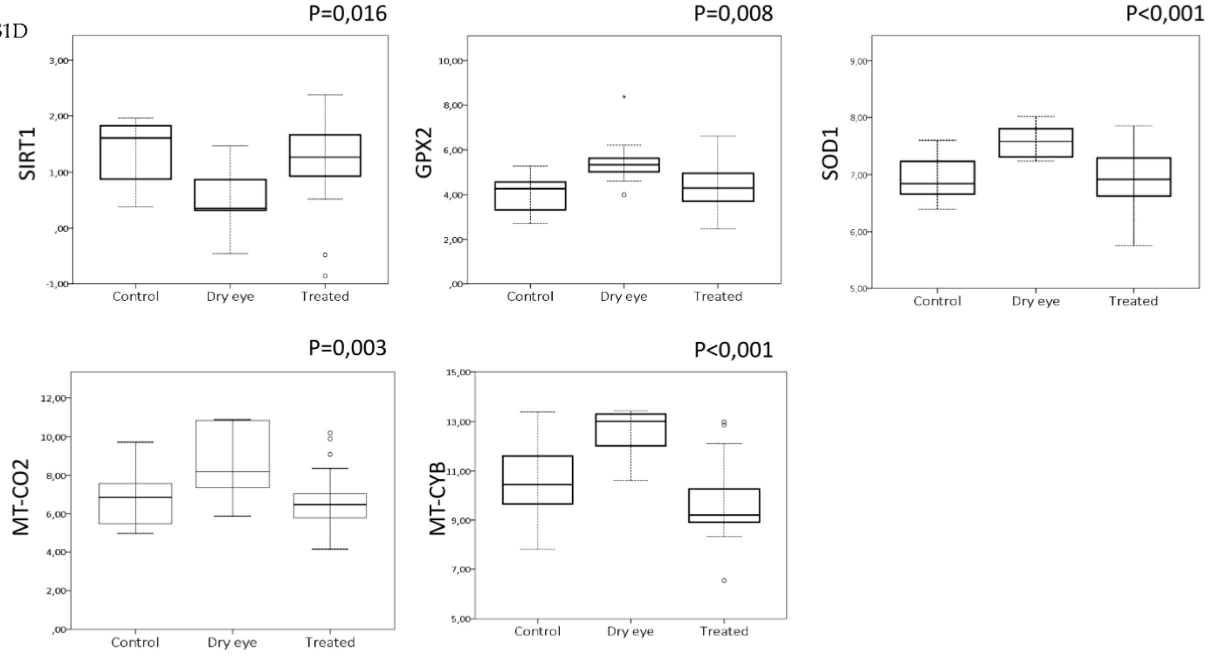

Figure S1. This figure shows gene expression levels dysregulated in dry eye syndrome patients related to the different pathways mentioned in the manuscript (immune system, cellular proliferation and differentiation, mitochondrial dysfunction, cellular movement and integrity and wound healing). A, Hippo signaling pathway-related gene expression. B, The TGF-b signaling pathway-related gene expression. C, Gene expression related to the integrin signaling pathway. D, Gene expression regulating mitochondrial metabolism and redox equilibrium. These genes were obtained in the RNA sequencing.

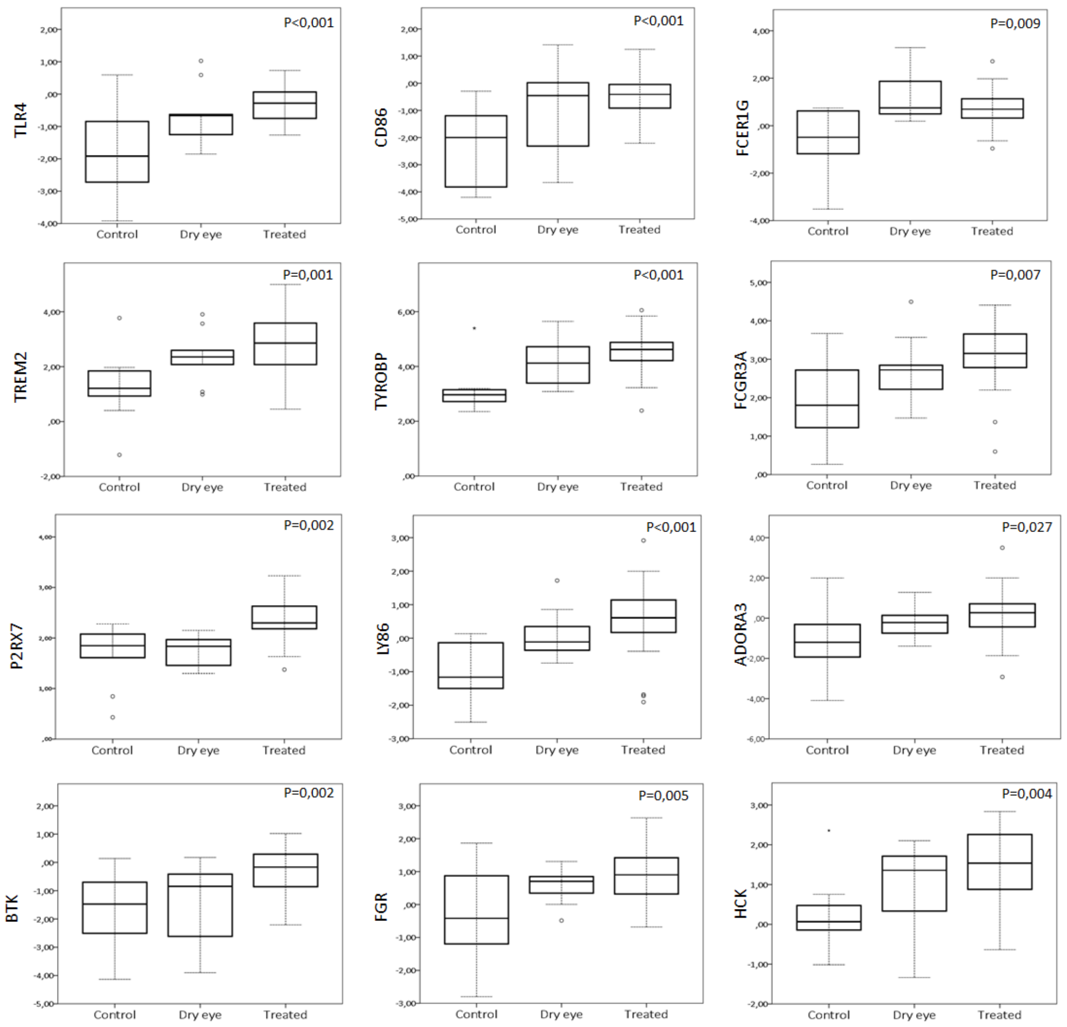

Figure S2. This figure shows gene expression levels dysregulated in glaucoma treated patients related to the different pathways mentioned in the manuscript (host defense and immune system, cellular proliferation and differentiation). These genes were obtained in the RNA sequencing.

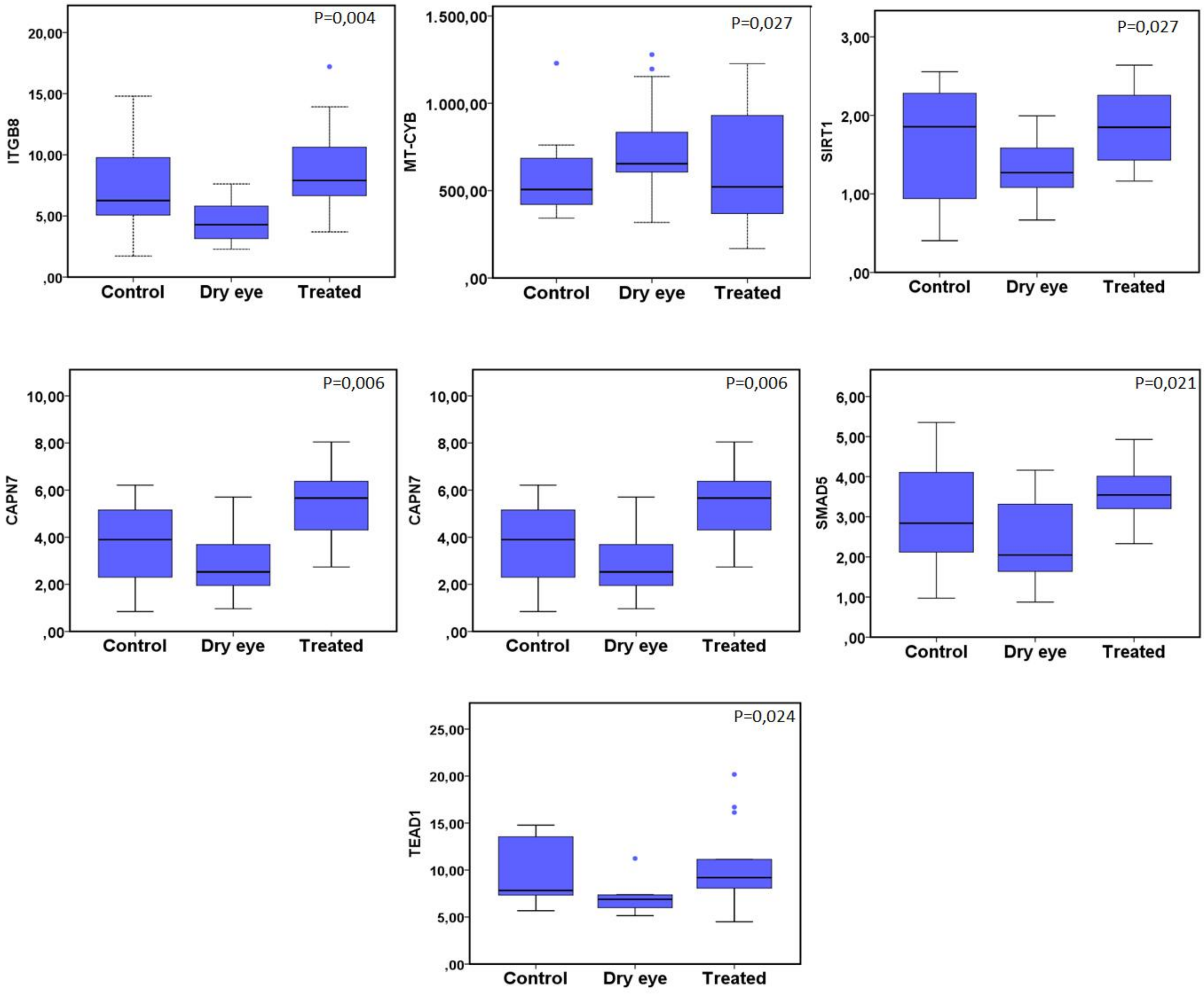

Figure S3. Validation of the gene expression in CIC samples from the dry eye syndrome patient group. 30 CIC samples out of 140 total CIC were selected for RNA validation.

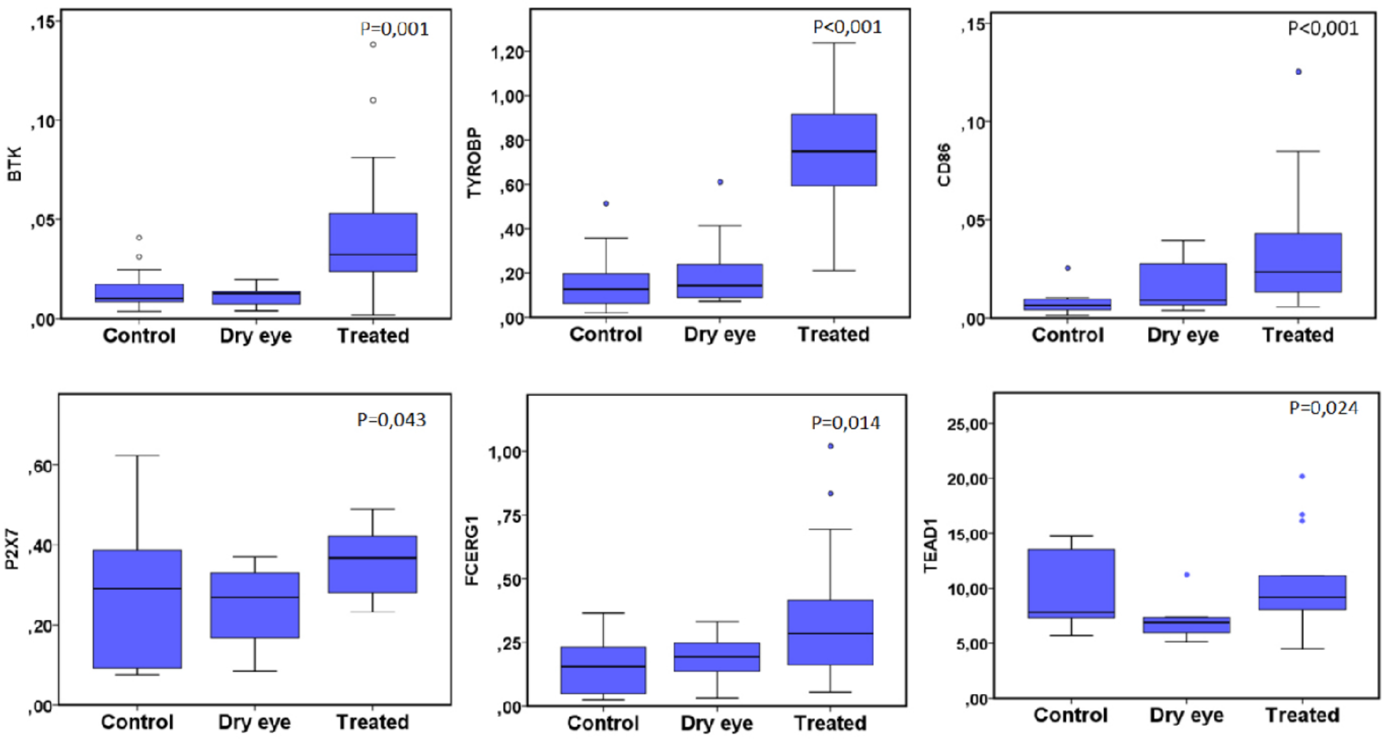

Figure S4. Validation of the gene expression in CIC samples from the treated glaucoma patient group. 30 CIC samples out of 140 total CIC were selected for RNA validation.
